# Obesity is associated with postoperative outcomes in patients undergoing cardiac surgery: a cohort study

**DOI:** 10.1186/s12871-022-01966-1

**Published:** 2023-01-04

**Authors:** Xiaofeng Jiang, Jianbo Xu, Shuai Zhen, Yanhong Zhu

**Affiliations:** 1 Department of Anesthesiology, Affiliated Jinhua Hospital, Zhejiang University School of Medicine, Jinhua, China; 2 Department of Anesthesiology, The First People’s Hospital of Pinghu, 500 Sangang Road, Danghu Street, Zhejiang 314200 Pinghu, China

**Keywords:** Obesity, Postoperative outcomes, Cardiac surgery

## Abstract

**Background:**

The purpose of present study was to determine whether obesity was associated with increased adverse outcomes after cardiac surgery.

**Methods:**

This is a retrospective cohort study from a large international database called the Medical Information Mart for Intensive Care III (MIMIC-III). Patients who underwent cardiac surgery and greater than 18 years old were divided into either nonobese (BMI < 30 kg/m^2^) or obese (BMI ≥ 30 kg/m^2^). The primary outcome of this study was 28-day mortality from the date of operation. Secondary outcomes included ICU mortality, 1-year mortality, incidence of postoperative atrial fibrillation (POAF), hospital length of stay (HOS_LOS) and ventilation-free days within 28 days (VFD_28).

**Results:**

Multivariate logistic regression analysis revealed a negative effect of obesity on 28-day mortality, with an adjusted odds ratio (OR) of 1.57 (95% CI 1.14–2.16; *p* = 0.005). The association remained significant when PSM analysis and double robust analysis with all covariates were performed. In terms of 28-day mortality, the mediating effect of longer ventilation duration on obese patients was noticeable, and the proportion of the effect mediated was 8.2% (95% CI 2.1–25.5%; *p* = 0.012).

**Conclusions:**

Among patients with cardiac surgery, obesity is associated with higher 28-day mortality. The longer ventilation duration may have mediated this effect. In future, considering the elevated incidence of the obese patients undergoing cardiac surgery, obesity stat should be included as one of the predictive variables for stratification of perioperative death risk.

**Supplementary Information:**

The online version contains supplementary material available at 10.1186/s12871-022-01966-1.

## Background

According to the World Health Organization, obesity prevalence is increasing worldwide [1]. Furthermore, obesity is a well-established risk factor for cardiovascular disease and its associated complications, such as heart failure, hypertension, or diabetes [2]; consequently, cardiac surgery has become widespread in obese patients, particularly in severely obese individuals [3, 4]. Previous studies have suggested that obese patients have worse clinical outcomes after cardiac surgery [5–7], however, other studies have found that obesity can be a positive factor. Obese patients have fewer problems and deaths after cardiac surgery than normal or underweight patients, in what is known as the “obesity paradox” [8–10]. These conflicting conclusions indicate that the relationship between obesity and the adverse outcomes of cardiac surgery remains uncertain.

Thus, the purpose of the present study was to determine whether obesity was associated with increased adverse outcomes after cardiac surgery. We hypothesized that obesity would have no paradoxical effect on postoperative outcomes.

## Methods

### Study design and participants

A large international database called the Medical Information Mart for Intensive Care III (MIMIC-III) [11] provided data in this a retrospective cohort study. The MIMIC-III is a large, single-center database comprising information relating to over 50,000 patients admitted to critical care units at the Beth Israel Deaconess Medical Center between 2001 and 2012. Data includes vital signs, medications, laboratory measurements, observations and notes charted by care providers, fluid balance, procedure codes, diagnostic codes, imaging reports, hospital length of stay, survival data, and more. By completing a test and earning certification, author Jiang was accountable for extracting data (certification number: 9,322,422).

Patients who underwent cardiac surgery in the MIMIC-III were eligible for inclusion. Cardiac surgery mainly concluded coronary artery bypass graft (CABG), valve surgery, pericardium surgery, septa surgery and thoracic aorta surgery. Patients who were under the age of 18 or missing BMI data were excluded. Furthermore, we analyzed only patient’s first ICU admission.

### Variable extraction

Structured Query Language (SQL) was used to obtain preoperative baseline characteristics, such as age, sex, BMI, ethnicity, admission type, surgery type, and comorbidities. Comorbidities including hypertension, diabetes, congestive heart failure (CHF), chronic pulmonary condition, stroke, renal disease, liver condition, cancer, coagulopathy and anemia were identified based on recorded ICD-9 codes.

Patients were classified as either nonobese (BMI < 30 kg/m^2^) or obese (BMI ≥ 30 kg/m^2^) according to the World Health Organization classification [1].

### Clinical outcomes

The primary outcome of this study was 28-day mortality from the date of operation. Secondary outcomes included ICU mortality, 1-year mortality, incidence of postoperative atrial fibrillation (POAF), hospital length of stay (HOS_LOS) and ventilation-free days within 28 days (VFD_28).

### Statistical analysis

Continuous variables are presented as the means and standard deviations, and values are presented as total numbers and percentages for categorical variables. Student’s t test was used to compare continuous data, the X^2^ or Fisher’s exact test was used to compare categorical data as appropriate. To avoid bias caused by missing data, the analysis was conducted after multiple imputations.

Multivariate regression was selected to evaluate the correlation between obesity and the clinical outcome. Baseline variables including age, sex, ethnicity, admission type, surgery type, and comorbidities were entered into a multivariate regression model as covariates. These variables were selected because of their clinical relevance. We used a stepwise backward elimination technique with a *p* < 0.05 to build our final models.

Sensitivity analyses, including propensity score matching (PSM) [12] and doubly robust analysis with all confounders [13, 14] — the combination of the PSM model and multivariate logistic regression analysis, were applied to evaluate the robustness of the primary findings of the study. When estimating the propensity scores of the patients, a multivariate logistic regression model was employed. A 1:1 nearest neighbor matching and a caliper width of 0.01 was applied in this study.

Causal mediation analysis (CMA) [15] is a technique for partitioning the total effect of an intervention into direct and indirect effects. The mediator mediates the indirect effect on the outcome. The analysis included average causal mediating effects (ACME), average direct effects (ADE), and total effects. In present study, we used CMA to investigate if the effect of obesity on the major outcome is partially mediated by the ventilation duration.

All statistical analyses were performed using the SPSS version 22.0 (IBM, Armonk, NY, USA) and EmpowerStats software (http://www.empowerstats.com), *p* < 0.05 were considered significant.

## Results

### Baseline characteristics

**Fig. 1:**
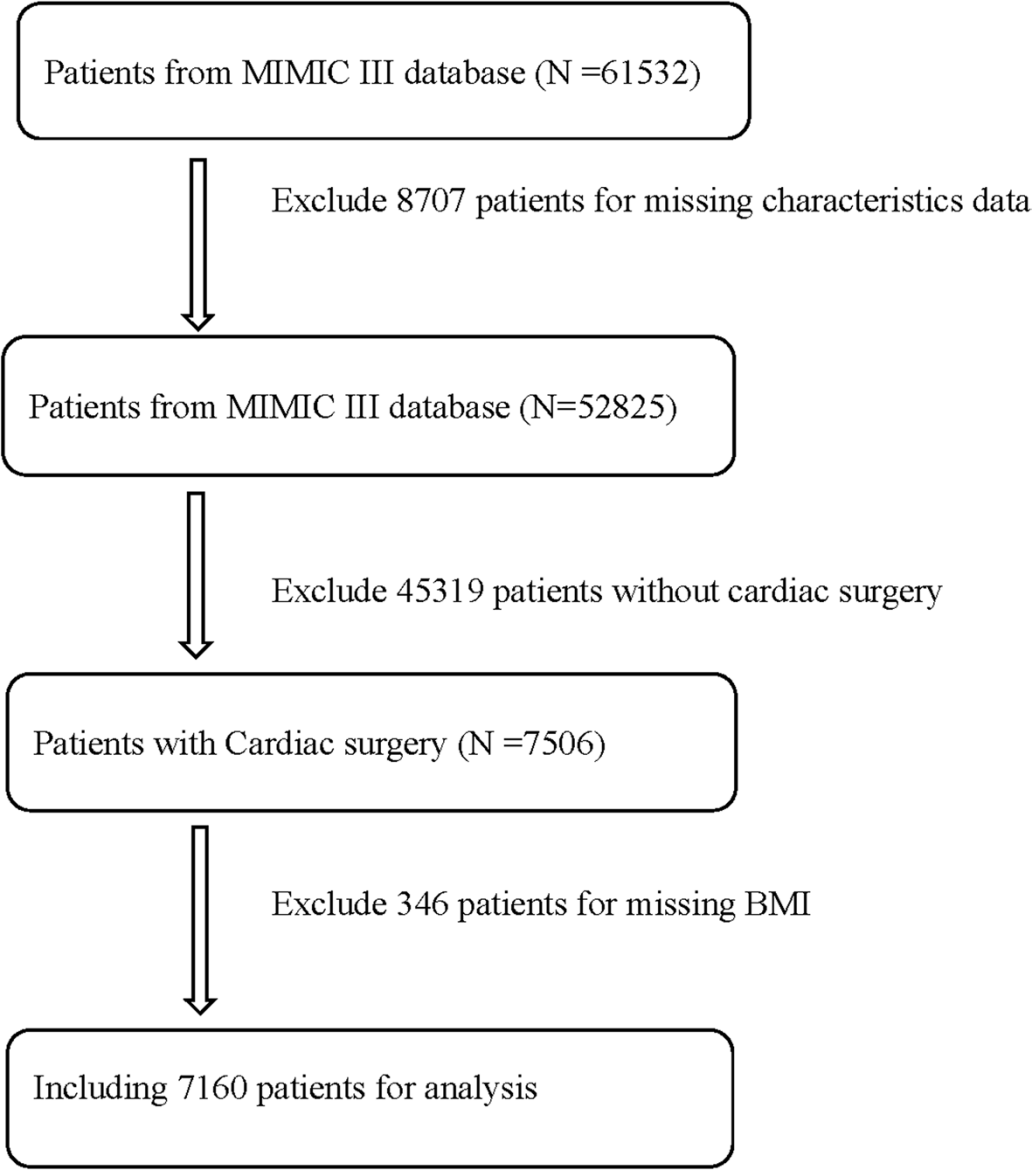
Patients selection and exclusion process

A total of 7160 patients with cardiac surgery were enrolled in present study. Figure 1 depicts the study selection process. Among the selected patients, 4737 patients had their BMI < 30 kg/m^2^, and 2423 patients had their BMI ≥ 30 kg/m^2^. Table 1 summarizes the baseline characteristics of the nonobese and obese groups. Compared with nonobese patients, obese people were more likely to with higher prevalence of hypertension, diabetes, chronic pulmonary condition, and coagulopathy.

**Table 1:** Comparisons of Demographics According to Body Mass Index Category before Matching

Baseline characteristics	BMI<30 (*n* = 4737)	BMI≥30 (*n* = 2423)	*p* value
Age, years	67.23 ± 12.64	64.09 ± 11.21	<0.001
Gender			
Male	3262 (68.86%)	1627 (67.15%)	0.140
Female	1475 (31.14%)	796 (32.85%)	
Ethnicity			
White	3359 (70.91%)	1760 (72.64%)	<0.001
Black	117 (2.47%)	79 (3.26%)	
Asia	69 (1.46%)	7 (0.29%)	
Other	1192 (25.16%)	577 (23.81%)	
Admission type			
Selective	2158 (45.56%)	1099 (45.36%)	0.915
Emergency	2393 (50.52%)	1233 (50.89%)	
Urgent	186 (3.93%)	91 (3.76%)	
Surgery type			
CABG	2317 (48.91%)	1370 (56.54%)	<0.001
Valve	1023 (21.60%)	445 (18.37%)	
CABG+Valve	613 (12.94%)	269 (11.10%)	
Pericardium	452 (9.54%)	206 (8.50%)	
Septa	177 (3.74%)	70 (2.89%)	
Thoracic aorta	155 (3.27%)	63 (2.60%)	
Comorbidities			
Hypertension	3155 (66.60%)	1785 (73.67%)	<0.001
Diabetes	1200 (25.33%)	1042 (43.00%)	<0.001
CHF	1360 (28.71%)	686 (28.31%)	0.724
Chronic pulmonary condition	686 (14.48%)	414 (17.09%)	0.004
Stroke	265 (5.59%)	138 (5.70%)	0.860
Liver condition	98 (2.07%)	54 (2.23%)	0.657
Renal failure	431 (9.10%)	231 (9.53%)	0.548
Cancer	139 (2.93%)	56 (2.31%)	0.125
Coagulopathy	425 (8.97%)	175 (7.22%)	0.011
Anemia	787 (16.61%)	395 (16.30%)	0.737

Data are Mean±SD or N (%)

*CABG* Coronary artery bypass graft, *CHF* Congestive heart failure

### Primary outcome

**Fig. 2:**
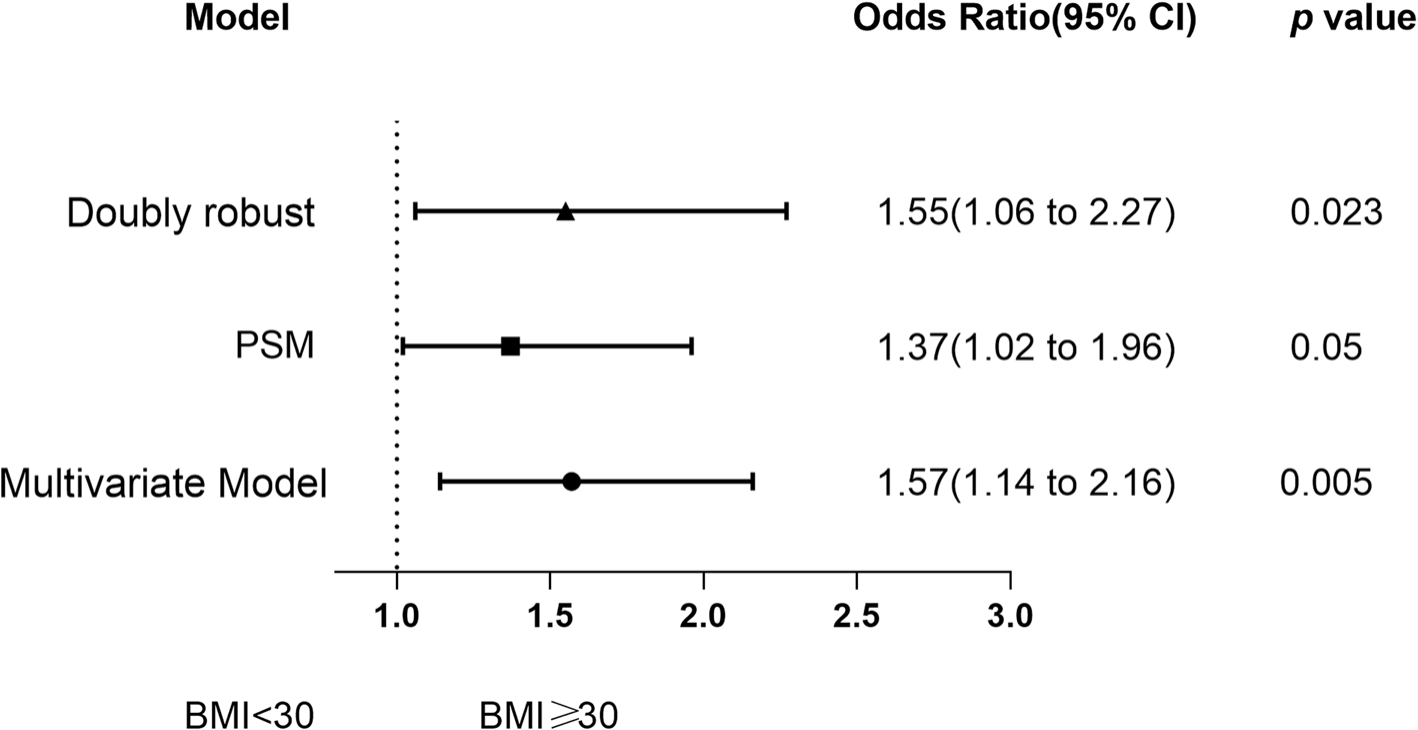
Association between obesity and 28-day mortality. The odds ratio represents the odds of death increase in obese patients compared to nonobese. PSM, propensity score matching

Multivariate logistic regression analysis revealed a negative effect of obesity on 28-day mortality, with an adjusted odds ratio (OR) of 1.57 (95% CI 1.14–2.16; *p* = 0.005) (Fig. 2). Using a 1:1 matching algorithm, 2371 patients in the nonobese group were matched with 2371 patients in the obese group after PSM (Table S1). The association remained significant when PSM analysis and double robust analysis with all covariates were performed (Fig. 2, OR 1.37, 95% CI 1.02–1.96, *p* = 0.05; OR 1.55, 95% CI 1.06–2.27, *p* = 0.023, respectively).

To investigate the effect of morbid obesity in cardiac surgery patients, additional analyses that classified obesity into overweight, class 1 and class 2–3 found an elevated risk of 28-day death in obese class 2–3 (Table 2). Subgroup analyses were also conducted to determine the relationship between obesity and the primary outcome according to the surgery type (valve surgery, CABG and combined surgery), and the results were presented in Table S2.

**Table 2:** Multivariable analyses of obesity status and 28-day mortality

Variables	OR	95% CI	*P*-value
Obesity Status			
Obesity Class 2–3	Ref.		
Obesity Class 1	0.79	0.48 to 1.29	0.341
Overweight	0.50	0.31 to 0.79	0.003
Normal weight	0.62	0.39 to 0.99	0.044

Ref. Reference group, World Health Organization classification of obesity class 2–3: BMI ≥ 35 kg/m^2^; Obesity Class 1: 30 kg/m^2^ ≤ BMI < 35 kg/m^2^; Overweight: 25 kg/m^2^ ≤ BMI < 30 kg/m^2^; Normal weight: 18.5 kg/m^2^ ≤ BMI < 25 kg/m^2^

*OR* Odds Ratio, *CI* Confidence Interval

### Secondary outcomes

**Fig. 3:**
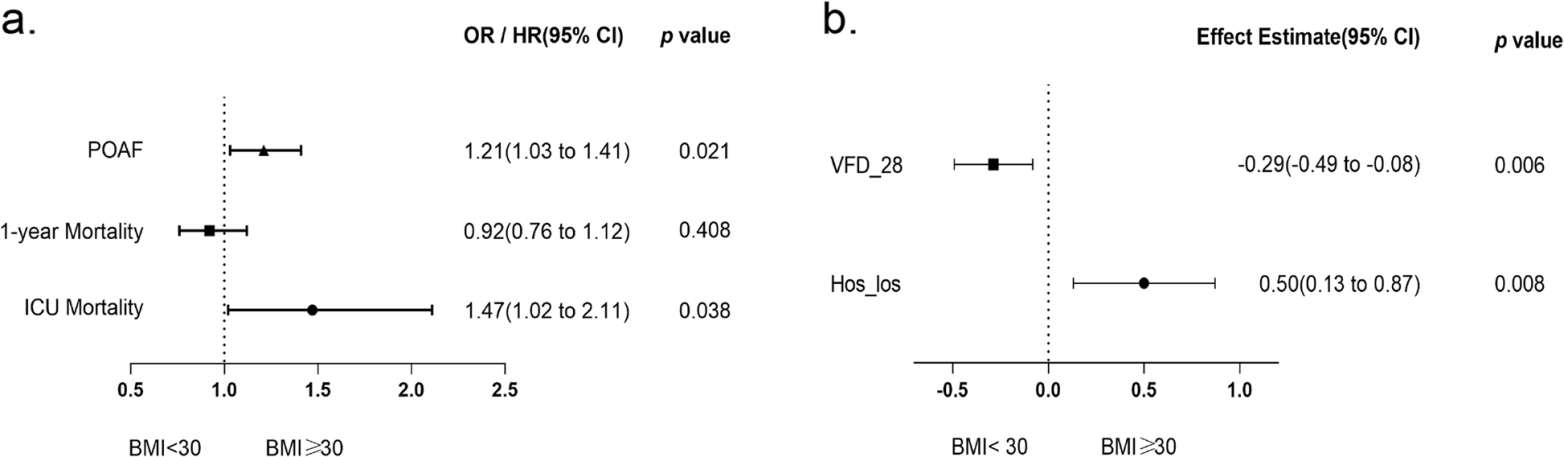
Association between obesity and secondary outcomes. **a** Odds ratio represents the odds of death or POAF in obese patients compared to nonobese. **b** Effect estimates and 95% confidence interval from the multivariable linear regression for VFD_28 and Hos_los. Effect estimate refers to the change in the outcome variable of obese patients compared to nonobese. POAF: postoperative atrial fibrillation; VFD_28: Ventilator-free days at day 28; Hos_los: length of hospital stay

Figure 3 presents the detailed results of the secondary outcome multivariate regression. Obesity significantly increased the ICU mortality and incidence of POAF (Fig. 3a), prolonged hospital length of stay, and decreased the number of ventilator-free days within 28 days (Fig. 3b). However, the two groups did not differ significantly in terms of 1-year mortality.

### Causal mediation analysis (CMA)

**Fig. 4:**
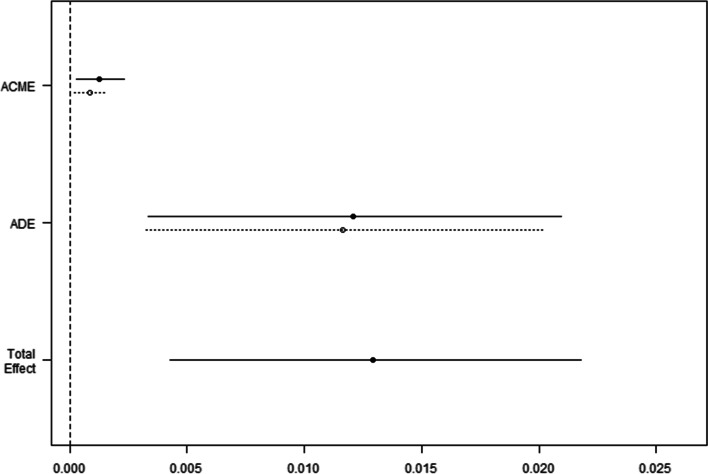
Causal mediation analysis for obesity in 28-day mortality. The solid line represents the obese group, and the dashed line represents the nonobese group

The direct and indirect effects of obesity on 28-day mortality were explored by CAM. When ventilation-free days within 28 days were employed as a mediator variable, the indirect effect was substantial. The total effect was 0.013 (95% CI 0.004–0.022; *p* = 0.002), the ACME was 0.001 (95% CI 0.0002–0.002; *p* = 0.02), the ADE was 0.012 (95% CI 0.003–0.021; *p* = 0.008), and the proportion of the effect mediated was 8.2% (95% CI 2.1–25.5%; *p* = 0.012) (Fig. 4). We concluded that obesity has a negative effect on 28-day mortality, which is mediated in part by a prolonged ventilation duration.

## Discussion

The results of this study indicate that obesity is related to a considerably higher 28-day mortality than non-obesity, as well as elevated ICU mortality and incidence of POAF, longer hospital length of stay, and lower ventilation-free days within 28 days, while there is no association with 1-year mortality. In terms of 28-day mortality, the mediating effect of a longer ventilation duration on obese patients was noticeable.

The increasing incidence of obesity and its accompanying health risks is a major public health issue worldwide. Specifically, excessive surgery is known to be associated with a BMI greater than 30 kg/m^2^ [16]. However, the EuroSCORE II model does not incorporate BMI as a risk factor for perioperative death stratification [17], as the risk of obesity in cardiac surgery remains controversial. Several studies [10, 18, 19] and a recent meta-analysis [9] have reported an “obesity paradox,” demonstrating a similar or even lower morbidity and mortality in obese patients than nonobese patients. Conversely, previous studies have also suggested higher morbidity and mortality rates in obese patients undergoing cardiac surgery. For instance, Kuduvalli et al. [20] reported a significantly prolonged ventilation duration and longer length of hospital stay in obese patients after CABG. Habib et al. [5] also concluded that obese patients experienced a greater incidence of morbidity and mortality following coronary artery bypass grafting. Our results are consistent with these findings, and refute the “obesity paradox” in terms of postoperative outcomes. The increase in 28-day mortality in obese patients remained significant after PSM and doubly robust analyses.

There are a number of reasons for why “obesity paradox” was observed in some cohort studies. Patients who are leaner may exhibit health behaviors such as smoking, frailty, comorbidities, or even cachexia, such as cancer. This leaner body habitus or a higher burden of comorbidities will independently contribute to increased morbidity and mortality. However, these health behaviors and comorbidities were considered in the multivariable model. Selection bias may be the most important explanation for “obesity paradox”. Risk stratification was adequately assessed for elective cardiac surgeries. Those with a high BMI but no metabolic syndrome are more likely to be deemed surgical candidates than those at a higher risk of obesity consequences [21]. The data of the present study were provided by MIMIC, which comprises not only elective surgery but also emergency and urgent surgery.

The present study indicates that obesity was not associated with 1-year mortality. Obesity may not be a risk factor for long-term survival due to renin–angiotensin responses, cytokine and neuroendocrine profiles, and differences in the pathogenesis of cardiovascular disease in obese and non-obese patients [22, 23]. Furthermore, changes in health habits after cardiac surgery may also affect long-term survival. Therefore, it is unclear whether the association between obesity and long-term mortality remains significant.

A mismatch between ventilation and perfusion, reduced functional residual capacity, and low respiratory reserve are more likely to occur in obese patients [16, 23]. By performing CMA, we observed that the effect of obesity on 28-day mortality in cardiac surgery patients was partially attributable to the prolonged ventilation duration.

Numerous limitations should be considered in this investigation. First, this study was performed using MIMIC databases spanning more than ten years, and we were unable to ascertain the exact year of patient admission. It is possible that changes in medical therapy and surgical techniques had an impact on the outcomes. Second, our study included a significantly higher proportion of non-obese patients than obese patients. Hence, we performed PSM to evaluate the robustness of the study’s primary findings in the sensitivity analysis. In addition, when separating obesity status to investigate different classes on the prognosis, the present study did not consider the underweight cohort (BMI < 18.5 kg/m^2^) due to the lack of patients (*n* = 96). Finally, the effect of metabolic syndrome [24, 25] on stratification of obesity based on fat distribution was not considered in the present study. Nonetheless, BMI continues to be a key starting point for patient categorization and comparisons with other studies, despite its limitations as a measure of obesity.

## Conclusion

Among patients undergoing cardiac surgery, obesity was associated with higher 28-day mortality rates. A longer duration of ventilation may have mediated this effect. A major part of pre-surgery counseling has focused on the influence of comorbidities and surgery risk, such as in the EuroSCORE II model. In the future, considering the elevated incidence of obese patients undergoing cardiac surgery, obesity status should be included as a predictive variable for stratification of perioperative death risk.

## Supplementary Information

**Additional file 1:** **Table S1.** Comparisons of Demographics According to Body Mass Index Category after Matching.

**Additional file 2:** **Table S2. **Multivariable analyses of obesity and 28-day mortality according to surgery type.  

## Data Availability

The datasets of the current study are available from the corresponding author on reasonable request.

## Abbreviations

CABG: coronary artery bypass graft
SQL: Structured Query Language
BMI: body mass index
CHF: congestive heart failure
POAF: postoperative atrial fibrillation
ICU: intensive care unit
LOS: length of stay
VFD_28: Ventilator-free days at day 28
PSM: propensity score matching
CMA: Causal mediation analysis
ACME: average causal mediating effects
ADE: average direct effects
