# Supplementary material for: Obesity is associated with postoperative outcomes in patients undergoing cardiac surgery: a cohort study
Source: BMC Anesthesiol. 2023 Jan 4;23:3. doi: 10.1186/s12871-022-01966-1 (PMC9811698; doi:10.1186/s12871-022-01966-1)
Supplement: Supplementary file 1 — Additional file 1: Table S1. Comparisons of Demographics According to Body Mass Index Category after Matching. [file 12871_2022_1966_MOESM1_ESM.docx]

| Table S1. Comparisons of Demographics According to Body Mass Index Category after Matching | | | |
| --- | --- | --- | --- |
| Baseline characteristics | BMI<30  (*n* = 2371) | BMI≥30  (*n* = 2371) | *p* value |
| Age, years | 64.81 ± 13.06 | 64.24 ± 11.20 | 0.109 |
| Gender  Male  Female | 1591 (67.10%  780 (32.90%) | 1597 (67.36%)  774 (32.64%) | 0.853 |
| Ethnicity  White  Black  Asia  Other | 1720 (72.54%  74 (3.12%)  36 (1.52%)  541 (22.82%) | 1715 (72.33%)  75 (3.16%)  6 (0.25%)  575 (24.25%) | <0.001 |
| Admission type  Selective  Emergency  Urgent | 1092 (46.06%)  1182 (49.85%)  97 (4.09%) | 1077 (45.42%)  1204 (50.78%)  90 (3.80%) | 0.753 |
| Surgery type  CABG  Valve  CABG+Valve  Pericardium  Septa  Thoracic aorta | 1239 (52.26%)  508 (21.43%)  256 (10.80%)  218 (9.19%)  83 (3.50%)  67 (2.83%) | 1326 (55.93%%)  441 (18.60%)  269 (11.35%)  202 (8.52%)  70 (2.95%)  63 (2.66%) | 0.080 |
| Comorbidities  Hypertension  Diabetes  CHF  Chronic pulmonary condition  Stroke  Liver condition  Renal failure  Cancer  Coagulopathy  Anemia | 1696 (71.53%)  936 (39.48%)  685 (28.89%%)  407 (17.17%)  144 (6.07%)  58 (2.45%)  222 (9.36%)  56 (2.36%)  197 (8.31%)  421 (17.76%) | 1736 (73.22%)  991 (41.80%)  678 (28.60%)  403 (17.00%)  133 (5.61%)  51 (2.15%)  228 (9.62%)  55 (2.32%)  174 (7.34%)  385 (16.24%) | 0.194  0.104  0.822  0.877  0.496  0.498  0.766  0.923  0.214  0.164 |
| Data are Mean±SD or N (%)  CABG: coronary artery bypass graft; CHF: congestive heart failure | | | |
