# Supplementary material for: Obesity is associated with postoperative outcomes in patients undergoing cardiac surgery: a cohort study
Source: BMC Anesthesiol. 2023 Jan 4;23:3. doi: 10.1186/s12871-022-01966-1 (PMC9811698; doi:10.1186/s12871-022-01966-1)
Supplement: Supplementary file 2 — Additional file 2: Table S2. Multivariable analyses of obesity and 28-day mortality according to surgery type. [file 12871_2022_1966_MOESM2_ESM.docx]

Table S2. Multivariable analyses of obesity and 28-day mortality according to surgery type.

| Variables | OR | 95% CI | P-value |
| --- | --- | --- | --- |
| CABG  Valve  CABG+Valve | 2.67  0.96  1.58 | 1.53 to 4.64  0.41 to 2.28  0.70 to 3.41 | 0.001  0.93  0.28 |

OR, Odds Ratio; CI, Confidence Interval

CABG: coronary artery bypass graft;
